# Medical Society of the County of Erie

**Published:** 1906-02

**Authors:** Franklin C. Gram


### SOCIETY PROCEEDINGS.

### Medical Society of the County of Erie

### Eighty-fifth Annual Meeting

Reported by FRANKLIN C. GRAM, M. D., Secretary.

DR. JOHN D. MACPHERSON, President, called the 85th annual meeting of the Medical Society of the County of Erie to order at 10:30 A. M., Tuesday, January 9, 1906, in the rooms of the Society of Natural Sciences, Buffalo Library Building.

The secretary read the minutes of the previous meeting, which were approved.

Dr. William Warren Potter, chairman of the membership committee, proposed the names of Dr. C. E. Abbott and Dr. Harry R. Frick for membership, upon which recommendation they were elected.

Dr. Henry R. Hopkins made a verbal report of the committee on legislation, relative to the work performed by this committee in the matter of securing a single State Medical examining board, as stated in the resolution introduced at the June meeting. He quoted the actions taken by various societies and individuals, as also of conferences of interested bodies. He offered the following resolution::

Resolved, That the Medical Society of the County of Erie hereby renews and reaffirms its positive conviction in the desirability of having in the state of New York a United Medical profession, united in a single State Medical Society, and united in a single County Medical Society in each of the several counties of the state. Resolved, That the united medical profession herein contemplated does not presume uniformity of medical practice or preclude the existence of special societies, local or state for special purpose scientific, philanthropic, or social, but has in mind the more general public purposes such as the elevation of the standard of medical education, the protection of the public health and such other general purposes for which medical societies have been found useful.

Resolved, That it is the confirmed conviction of this society that for the accomplishment of the above mentioned desideratum the agitation and advocacy for a single State Examining Board should be encouraged and continued. Resolved, That this matter be referred to the Committee on Legislation with authority and discretion to continue its work.

Dr. J. B. Coakley moved the adoption of the report, and of the resolutions.

Dr. William Warren Potter asked if this was the report of the legislative committee. Receiving an affirmative answer he maintained that there was no regular standing committee known as the legislative committee, and none was appointed this year. A special committe was appointed in February, 1905 to go to Albany to oppose certain specific measures, but had no further power. He advised a reference of this matter to a committee on legislation to be appointed.

Dr. Hopkins said that at the semi-annual meeting in June this committee was given all contemplated powers, and quoted the resolutions adopted at that meeting.

Dr. William Warren Potter still maintained his position.

Dr. Hopkins rose to a point of order, stating that the minutes of the June meeting showed that such a committee existed, and was recognised as such by the society.

The motion was then adopted.

Dr. Hopkins asked if this carried with it the adoption of the resolutions offered by him.

The chair ruled that it did.

The president then appointed as nominating committee Drs. Ernest Wende, Delancy Rochester, and William Warren Potter.

This committee reported the following nominations. For President, Dr. A. H. Briggs; for Vice-President, Dr. Edward Clark; for Secretary, Dr. Franklin C. Gram; for Treasurer, Dr. Dewitt C. Greene; for Censors, Drs. H. R. Hopkins, DeLancy Rochester, Irving W. Potter, J. H. Grant and F. E. Fronczak; for legislative committee, Drs. A. G. Bennett, Wm. C. Krauss and Ernest Wende; for membership committee, Drs. William Warren Potter, C. A. Wall and A. T. Lytle.

Dr. William Warren Potter moved the adoption of the report.

Dr. Rochester moved an amendment that the report be adopted with the exception of the nominee for president, stating his reasons.

Dr. Coakley spoke against the amendment.

Dr. Walter D. Greene, Health Commissioner of Buffalo. explained the question raised by Dr. Rochester and hoped the amendment would not prevail.

Similar remarks were made by Dr. S. S. Green and Dr. A. A. Hubbell, all of the speakers showing the high esteem in which Dr. Briggs is held by the profession and the community.

Dr. Briggs rose for a personal explanation of the question raised. Under other circumstances he would promptly withdraw his name as a candidate, but under the present conditions he will meet the issue and leave the verdict with the society.

Dr. Rochester's amendment was lost. The report was then adopted and the secretary instructed to cast the ballot of the

Society for the nominees, which being done, they were declared duly elected.

Dr. Hopkins made a verbal report in which he reviewed the work of the board of censors, going minutely into the various details. He explained what was done in the cases of Cooper, Antonious, Schwabe, Darlington, the Vin Cure Remedy Co., and suggested that this society authorize the incoming board of censors to take cognisance of the relation of certain members of this society with said company, also that the society instruct the censors to further investigate the Woodward case. He said it should be made clear that we prosecute criminals not for the protection of the society, but for the protection of the public. He had been requested by the Board of Regents at Albany to make an investigation and report on the Atlantic School of Osteopathy, an institution which had graduated 198 students, and as a result of such investigation the president of the school was arrested. The school authorities were advised by their counsel that they were violating the state law and had no defense. The faculty was dispersed and the school broke up.

Dr. Hopkins submitted the report of the Society's Counsel, which was also made the report of the censors.

The report was adopted. Dr. Hopkins then offered the following resolutions:

Resolved, That the Medical Society of the County of Erie justly takes pleasure in making public acknowledgment of the invaluable services which District Attorney Edward E. Coatsworth Esquire, and Assistant District Attorney Abbott have ever given cordially and unstintingly to the work of the Censors.

Resolved, That it is the conviction of this society, that prevention of crime is one of the first obligations of the state and that medical criminals and their work should be an abomination and an abhorrance to all good citizens.

Resolved, That the Medical Society of the County of Erie is under great obligations to its overworked and underpaid counsel John Lord O'Brian, Esq., whose constant inspiration has made the work of the Censor possible.

The resolutions were adopted.

Dr. William Warren Potter moved a vote of thanks to Dr. H. R. Hopkins for his individual indefatigable work, especially in relation to the Atlantic School of Osteopathy.

This was adopted.

On motion of Dr. Krauss the powers asked for by the censor's report were given to the next Board of Censors.

And on motion of Dr. Coakley the censors were empowered to investigate the status of persons practising osteopathy in this county.

Dr. Dewitt C. Greene submitted his annual report as treasurer, which was referred to an auditing committee of Drs. Satterlee, Ward and Jolls, who reported it correct.

The trasurer read a communication from the counsel of the Society relative to the $250.00 fine, which he had not received. Dr. Hopkins explained what steps had been taken in this matter during the treasurer's absence from the city, and on motion of Dr. Hubbell the action of Dr. Hopkins was approved.

On motion of Dr. Hubell the Treasurer was directed to order the usual copies of the state society's Transactions, as per his communication.

On motion of the secretary, Drs. Dwyer and Callanan were appointed a committee to prepare a suitable memorial on the death of Dr. Walsh.

Dr. C. E. Congdon made a personal explanation of his connection with the Vin Cure Remedy Co., and how he became connected with it. When he learned the standing of the company, he withdrew.

The secretary stated that he had just received a certified list of members of the Erie County Medical Association, and the following communication (which, though dated January 3, had not been received until January 8th.) :

Dear Doctor:— I beg leave to call your attention to the enclosed resolutions passed at the meeting of the House of Delegates, of the Medical Society of the State of New York, held at Albany, December 14, 1905. Kindly bring these matters to the attention of your County Society, so that action can be taken thereon as soon as possible. If you have any doubt in regard to any of these resolutions, you can communicate with Dr. Wisner R. Townsend, Acting Assistant Secretary, 64 Madison Avenue, New York, who will advise you on the subject.

I will send copy of the tentative by-laws in a few days.

You are requested to place on your roster all names on the enclosed list of members of the County Association of your County, who, according to the Agreement, are now members of the Medical Society of the State of New York, of your County and District Branch.

Hoping to see you in Albany at the annual meeting, I am

Very truly yours, F. C. Curtis, Secretary.

Resolved, That the secretary of the Medical Society of the State of New York immediately notify the respective presidents and secretaries of each County Medical Society within the State of New York, that the order of consolidation of the Medical Society of the State of New York and The New York State Medical Association has been entered and filed, and direct them that in compliance with Section VI of the Agreement, which is made a part of the order of consolidation, each of such Medical Societies is hereby directed to call a meeting of all their members, including all members of the Association in good standing at the date of the consolidation, residing in the counties in which the meetings shall be held, respectively, for the purpose of effectuating the plan of organisation under the Constitution and By-Laws hereto annexed, and for the transaction of such other business as may come before the meeting, and that upon receipt of the enclosed certified copy of a list of members of the County Association in their county, such members shall be placed upon the list of County Societies without notice and without further action. (See paragraph third of the Agreement.)

Moved, seconded and carried:

Resolved, That the secretary notify the president and secretary of each County Association, in a county containing no County Society, that they shall forthwith change the name of such Association to Society, and forthwith effectuate the plan of organisation of County Societies in compliance with the State Society By-Laws.

Moved, seconded and carried:

Resolved, That the secretary be directed to send a copy of tentative By-Laws to the County Societies, with the statement that these By-Laws were made by a sub-committee of the Committee of Conference, and were advisory and suggestive only; that they can be adopted as they stand and would be perfectly acceptable to the Council; or that they could be amended, altered or used as a guide in making new By-Laws by the counties.

Resolved, That the Medical Society of the State of New York furnish to its members for the year 1906, the services of an attorney-at-law in actions brought for alleged malpractice, under certain conditions to be hereinafter provided, and that the president and acting assistant secretary be empowered to employ counsel to conduct the defense.

Resolved, That the Medical Directory be continued.

Moved, seconded and carried:

Resolved, That an assessment be placed upon each member of the Medical Society of the State of New York, at a uniform per capita rate of $3.00.

Moved, seconded and carried:

Resolved, That the County Societies be directed to levy such assessment and that the treasurers remit as promptly as possible to the State treasurer.

Moved, seconded and carried:

Resolved, That a Committee of Three be appointed by the chair to authorize and audit expenditures until further order of this body.

Moved, seconded and carried:

Resolved, That the secretary call the attention of officers of County Societies to the requirement of Chapter IX, Section 8, of the By-Laws of the Medical Society of the State of New York.

Each County Society may adopt a constitution and by-laws for the regulation of its affairs, provided that the same shall first be approved by the Council of this Society.

Moved, seconded and carried:

Resolved, That the secretary call attention of those who are to read papers at Albany, at the annual meeting, to Chapter X, Section 2, of the By-Laws of the Medical Society of the State of New York.

All papers read before the Society or any of its sections shall become the property of the Society. Each paper, or a copy thereof, shall be deposited with the secretary after the same shall have been read.

In pursuance to the agreement of the Joint Committee of Conference, it was moved, seconded and carried:

Resolved, That the referendum proposition relating to the Principles of Medical Ethics of Section 7 of said Agreement be and hereby is referred to a special committee of five members of which the president shall be one, with the power to issue the call for the vote at the earliest practical time, canvass and report the results of the vote on the proposition to the House of Delegates at its next meeting thereafter.

Moved, seconded and carried:

Whereas, Article IV of the Constitution of the American Medical Association states:

"Those State and Territorial Medical Associations which have, or which hereafter may, become organised in accordance with the general plan of organisation of the American Medical Association, and which have declared by resolution their allegiance to the said American Medical Association, and which shall agree with other State and Territorial Medical Associations to the formation and the perpetuation of the House of Delegates of the American Medical Association shall be recognised as Constituent Associations."

Resolved, That the Medical Society of the State of new York, by virtue of having conformed to the requirements, declares its allegiance to the American Medical Association, and thereby becomes its constituent society in the State of New York.

On motion of Dr. Wall the communication was received and filed.

Dr. C. A. Wall offered the following resolution:

Resolved, That the Medical Society of the County of Erie, an incorporate body, does hereby accept the agreement of consolidation and the new Constitution and By-Laws of the Medical Society of the State of New York, as binding upon it and thereby making it the component part of State Society for Erie County and that it shall consist of those members of the State Society resident in said County.

That the papers from the State Society be received and that Erie County accept the new rules of the State Society and resolve that the said Erie County Medical Society be the constituent part of the State Society resident in Erie County.

The resolution was adopted.

On motion of Dr. Hubbell, the president in his discretion, was authorised to call a special meeting of the Society, if deemed necessary after receiving a copy of the By-Laws..

Dr. Howe moved that those members who are present at the next annual meeting of the State Society at Albany, be given power to elect from among their number representatives to the House of Delegates.

The motion was adopted.

As the time occupied in trasacting the foregoing business had advanced to nearly 1:00 o'clock the president called for the pleasure of the Society relative to the scientific programme which had been prepared. On motion this was left for a future meeting.

The president-elect, Dr. A. H. Briggs, was then called to the chair and thanked the members for the confidence bestowed in him.

The society adjourned, subject to the call of the president.

